# Cerebral iodized lipid embolization via a pulmonary arteriovenous shunt: rare complication of transcatheter arterial embolization for hepatocellular carcinoma

**DOI:** 10.1186/1477-7819-11-122

**Published:** 2013-05-30

**Authors:** Zoltán Bánsághi, Pál Novák Kaposi, Gábor Lovas, Gyöngyvér Szentmártoni, György Várallyay, Pál Bata, Ildikó Kalina, Balázs Futácsi, Viktor Bérczi

**Affiliations:** 1 Department of Radiology and Oncotherapy, Semmelweis University, Budapest, Hungary; 2 Department of Neurology, Semmelweis University, Budapest, Hungary; 3 MRI Research Center, Szentágothai J. Knowledge Center, Semmelweis University, Budapest, Hungary; 4 Department of Radiology, St. Imre Hospital, Budapest, Hungary; 5 Department of Neurology, Jahn Ferenc Teaching Hospital, Budapest, Hungary

**Keywords:** Iodized lipid embolism, TAE, HCC, Right-to-left shunt

## Abstract

We report the first European case of cerebral iodized lipid embolism post transcatheter arterial embolization for hepatocellular carcinoma. Lipiodol emboli and corresponding multifocal brain ischemia were documented with computed tomography (CT) and magnetic resonance (MR) in the acutely symptomatic patient. Transcranial Doppler sonography with contrast indicated a right-to-left shunt, while on a follow-up CT scan lipiodol embolization was detected in both lungs. Dilated pulmonary vessels and thick vascular channels were seen in the vicinity of the right diaphragm suggestive of pulmonary arteriovenous shunt. The patient symptoms regressed with supportive care alone, but he died 5 months later due to hepatic failure unrelated to the procedure.

## Background

Hepatocellular carcinoma (HCC) is the fifth most common solid malignancy [1]. Early stage HCC can be curatively treated by liver resection, transplantation, radiofrequency ablation (RFA), or percutaneous ethanol injection (PEI) [2]. Unfortunately, in 60% to 70% of the patients, the tumor has already progressed into an advanced stage at the time of diagnosis; thus, only palliative treatment could be offered. Transcatheter arterial chemoembolization (TACE) has become the mainstay therapy for HCC patients ineligible for surgery or tumor ablation [2]. Transcatheter arterial embolization (TAE) when Lipiodol is injected alone has been extensively used and achieved comparable outcomes to TACE in clinical trials [3]. The overall complication rate of TACE/TAE is relatively low, 2.9%, but occasionally severe side effects may develop leading to death or debilitating neurological deficits [2,4].

Pulmonary arteriovenous (AV) shunts are often hereditary but can also be observed in a number of acquired conditions including trauma and hepatic cirrhosis as well as metastatic disease. In 95% of the cases, the pulmonary AV shunt originates from a pulmonary artery, and occasionally from a systemic artery [5]. In liver cirrhosis, multiplex pulmonary AV shunts can lead to hypoxemia and peripheral cyanosis consistent with the diagnosis of hepatopulmonary syndrome [6].

Previous reports have already suggested that pulmonary AV shunts are the likely source of non-target embolism post TAE/TACE [7]. In the present case, however, pulmonary embolism, right-to-left shunting, and a pulmonary AV shunt were simultaneously identified with radiographic studies in agreement with a previously hypothesized double shunt mechanism. According to our knowledge the present case is also the first CLE reported from a European country.

## Case presentation

**Figure 1:**
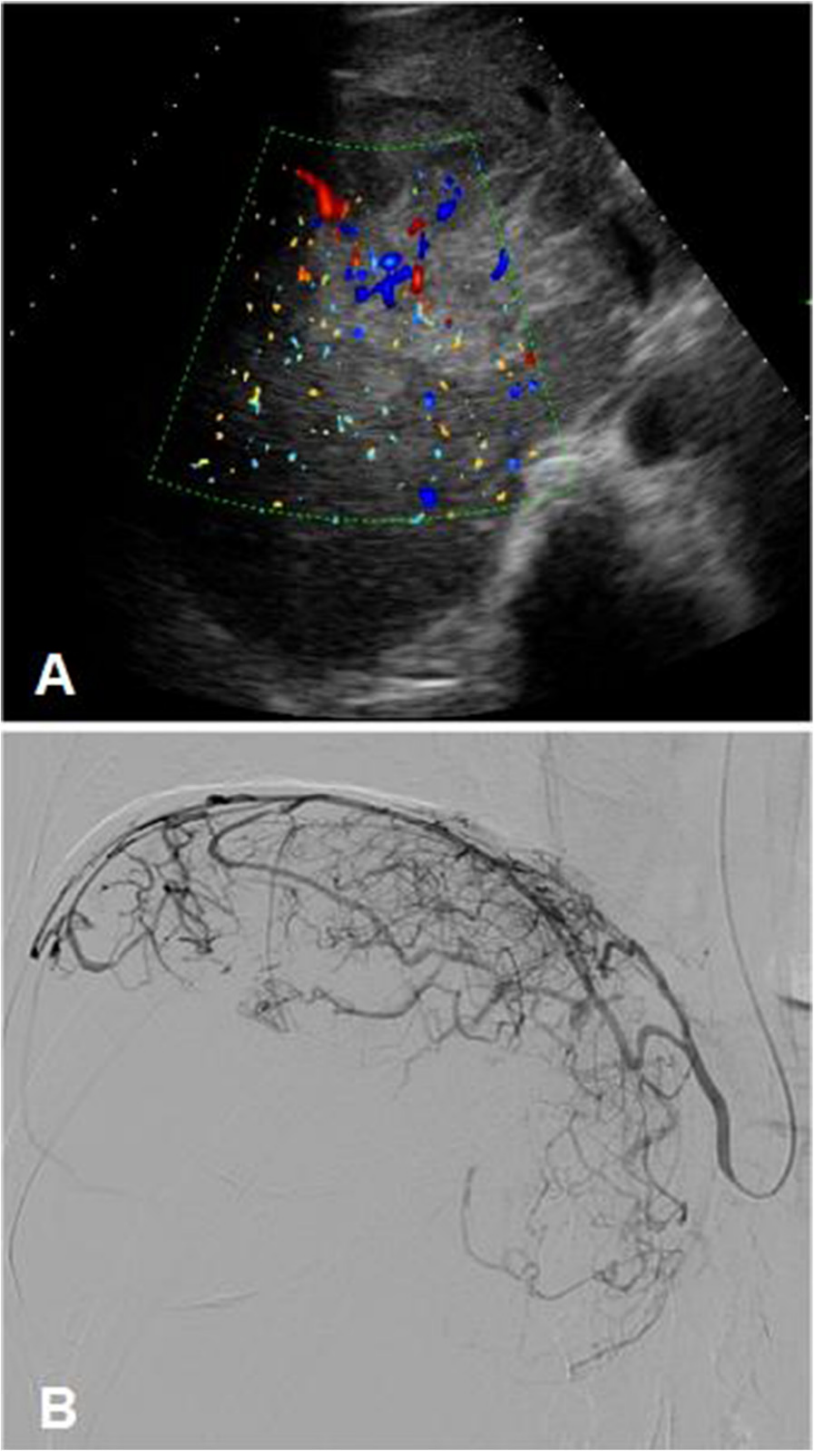
**The large right lobe HCC was embolized via the right inferior phrenic artery.** (**A**) Color Doppler sonography of the liver revealed a hypervascular HCC, which almost completely replaced the right lobe and extended to the diaphragm. (**B**) According to the initial angiogram the tumor was in part supplied by both the right inferior phrenic and the right hepatic arteries. Following selective catheterization and TAE the feeding arteries were closed off with gelatin sponge particles.

**Figure 2:**
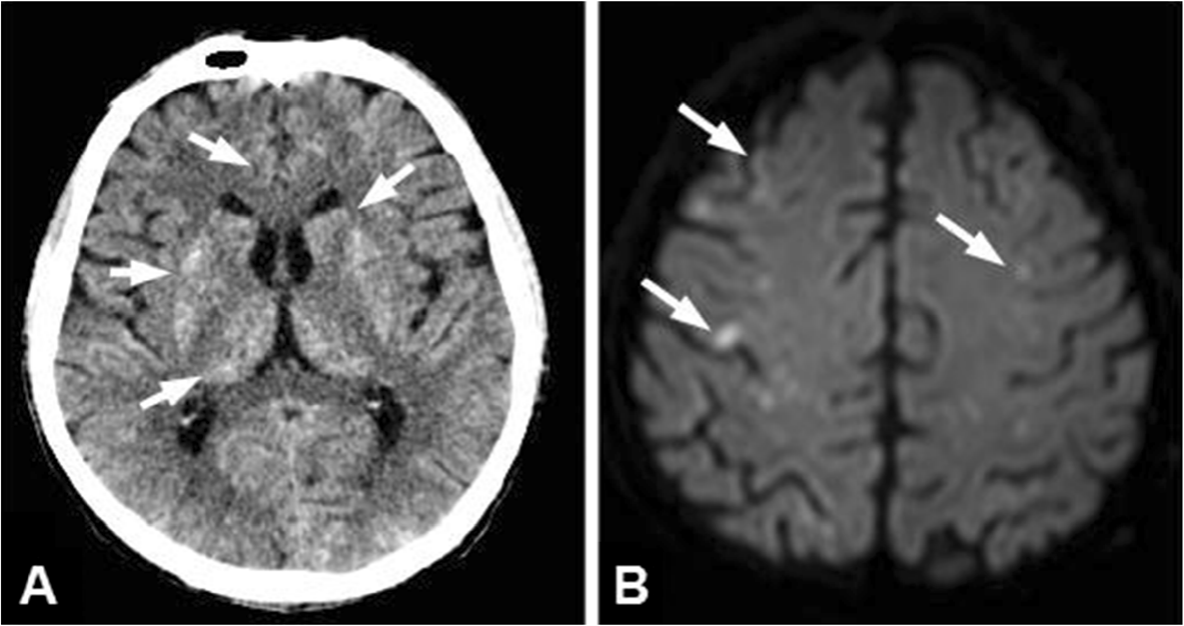
**Cerebral iodized lipid emboli were detected shortly after the embolization procedure.** Two hours post TAE the patient developed sudden neurological symptoms. (**A**) An emergent CT revealed scattered high attenuation spots in the gyruses and basal ganglia in both hemispheres (arrows) indicative of iodized lipid embolization. (**B**) Diffusion-weighted MRI on the next day found disseminated acute ischemic lesions (arrows) in corresponding locations.

**Figure 3:**
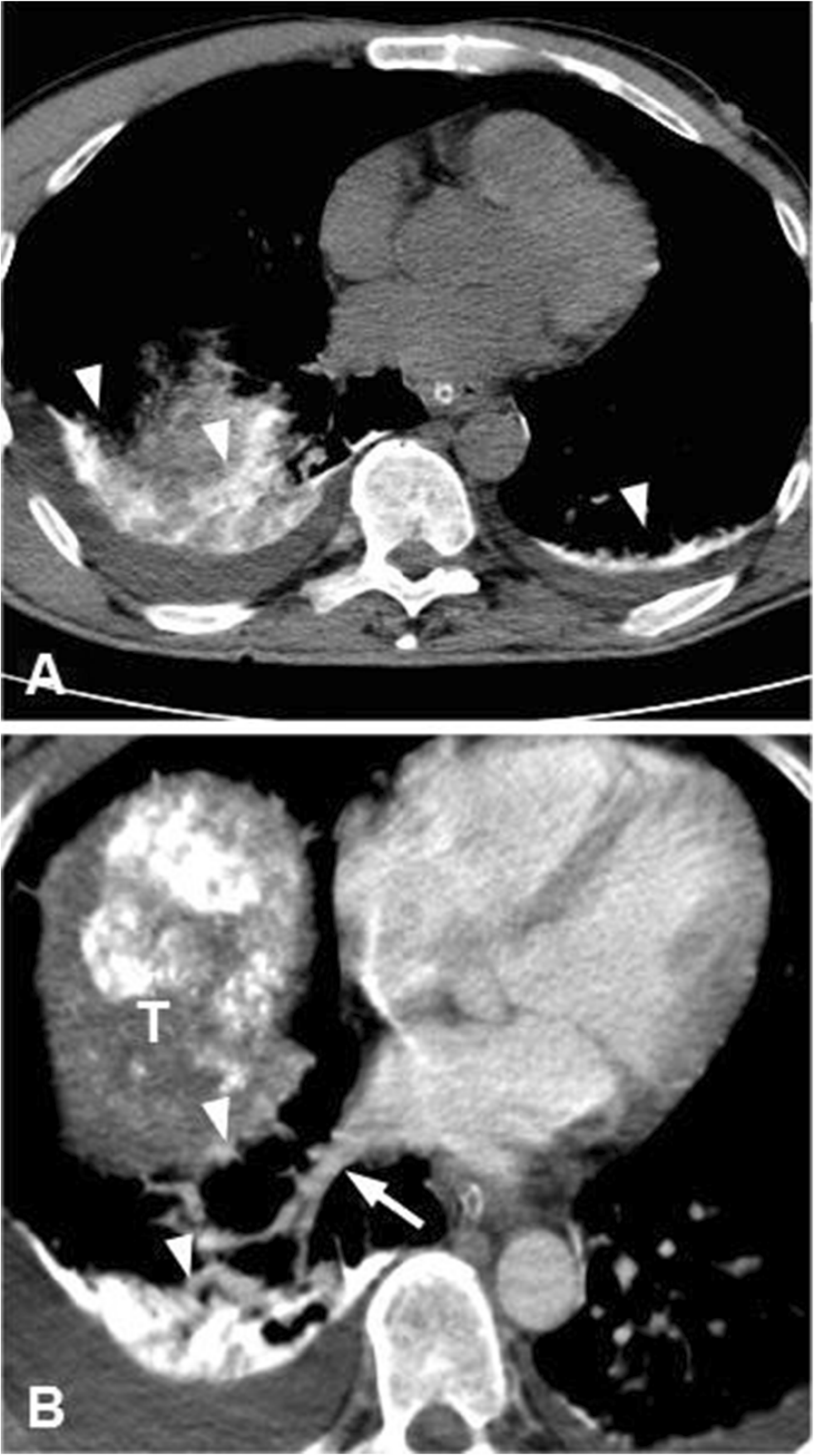
**Extensive pulmonary embolization and dilated pulmonary vessels could be observed post embolization.** (**A**) A follow-up CT performed 3 days post TAE showed extensive lipiodol embolization (arrowheads) in both lungs well visible in the atelectatic basal segments. (**B**) In the vicinity of the right diaphragmatic surface, widened vascular spaces (arrowheads) and a thick pulmonary vein (arrow) could be identified, suggestive of a pulmonary AV shunt.

A 52-year-old man with a history of alcohol abuse presented with right upper quadrant pain. On the abdominal ultrasound, a large, 18 × 16 cm hepatic mass was identified. The tumor replaced most of the right liver lobe and also expanded to the right diaphragm. Dilated vascular channels were detected inside the tumor on color Doppler sonography (Figure 1A). However, no spectral Doppler analysis was performed to identify potential AV shunts. Fine needle aspiration biopsy (FNAB) of the lesion verified the diagnosis of HCC. Subsequently, to reduce tumor size the patient was referred to a TAE. Angiogram from a left brachial artery puncture revealed that the liver mass was in part supplied by the right inferior phrenic artery (IPA) (Figure 1B) as well as by the right hepatic artery branching off from the superior mesenteric artery. The angiography did not detect any apparent high flow shunts inside the tumor, although small shunts might have gone undetected pre-embolization. The feeding arteries were selectively catheterized, embolization with 50 mL Lipiodol (Guerbet, France) was performed, and the arterial lumens were closed off with Spongostan (Johnson & Johnson, NJ, USA) sponge particles. Thirty minutes later the patient became agitated, disoriented, and unresponsive, and started shivering. Cyanosis of both lower extremities was observed. A non-contrast computed tomography (CT) scan obtained 2 h after the procedure demonstrated hyperdense (60–65 HU) lipid droplets scattered in the gyruses and the basal ganglia in both hemispheres (Figure 2A). The electroencephalogram (EEG) did not display abnormal activity compatible with epileptic seizure or hepatic encephalopathy, however, subcortical dysfunction was seen. TCD showed normal flow in the circle of Willis. Following venous injection of agitated saline solution, a RLS was detected by demonstrating microbubbles in the right middle cerebral artery. On the following day, diffusion-weighted magnetic resonance imaging (DW-MRI) of the brain showed disseminated areas of restricted diffusion indicative of recent ischemic injury (Figure 2B). On the time-of-flight MR angiography (TOF-MRA) all intracranial arteries were patent. No intracranial bleeding or vascular shunt was observed. On the second day post-embolization the patient became febrile, his status progressively deteriorated, myoclonus of the whole body and horizontal nystagmus were detected. The patient was transferred to the intensive care unit, where benzodiazepine sedation, antibiotics, and ventilation therapy were started. On a follow-up CT lipiodol embolization was found in both lungs (Figure 3A), which was especially apparent in the atelectatic basal areas. In addition, dilated vascular channels and a widened pulmonary vein (Figure 3B) could be identified next to the right diaphragmatic surface suggestive of a pulmonary AV shunt. Inside the liver mass, extensive tumor necrosis was detected. The patient’s respiratory and neurological status gradually improved in the next 3 to 4 weeks. Unfortunately, the patient died 5 months later because of hepatic failure unrelated to the procedure. The autopsy did not find macroscopic signs of brain ischemia or evidence of a septal defect.

## Discussion

Cerebral iodized lipid embolism is a relatively rare complication of TAE/TACE with <20 cases published in the literature thus far [7,8]. To our best knowledge, ours is the first case reported from a European country. This could be explained by higher chronic hepatitis infection rates and greater incidence of HCC in other regions [1]. Also, due to the better surveillance and advancements in patient management multiple embolizations can be performed [2]. Therefore, increase in the number of procedure-related adverse events can be expected. The clinical and radiographic presentation of CLE is similar to lipid embolization. In most of the reported cases, neurological deficits were mild or moderate in severity and showed significant improvement or complete remission within a few weeks [9]. Characteristically, patients develop acute symptoms during or shortly after the embolization procedure. Interestingly, Wu *et al.* reported a delayed case of cerebral embolism, which was observed 69 h following TACE [7]. Although most of the patients required supportive care alone, massive lipid embolization post TACE can occasionally result in a fatal outcome [4]. In our case, ventilation support and intensive therapy were necessary to stabilize the patient’s condition, in part because of the pulmonary embolization and tumor necrosis syndrome.

When CLE is suspected, radiographic studies can confirm non-target embolization by demonstrating embolization material in the brain parenchyma [9]. Lipiodol itself is a positive radiographic contrast agent. Therefore, on a non-enhanced CT iodized lipid droplets show up as scattered hyperattenuating dots in the basal ganglia, as well as in the cerebral and cerebellar hemispheres. As in the current case, MRI with diffusion-weighted imaging is helpful to identify concomitant ischemic lesions, but fat intensity from small lipid droplets could not be visualized. Residual ischemic lesions are seldom seen on follow-up scans (4). EEG was essential to exclude other causes of the abrupt neurological symptoms such as seizure or hepatic encephalopathy.

The exact embolization route often remains undetermined in the reported CLE cases. Our case is exceptional since we could also demonstrate that a pulmonary AV shunt was the likely source of the cerebral emboli. In the vicinity of the right diaphragmatic surface, dilated vascular channels and a widened pulmonary vein could be identified suggestive of a pulmonary shunt. This pulmonary AV shunt most likely was supplied by a segmental pulmonary artery branch. Consistently, right-to-left shunting was documented on the saline contrast TDS. Pulmonary AV shunts may develop on the tumor surface or at pleural adhesions. CLE is often described in HCC patients with diaphragmatic involvement or large right lobe tumors. In addition pulmonary embolism may have facilitated the opening of pre-existent pulmonary AV shunts by increasing the pulmonary arterial pressure [7]. Similar to our patient, at least six CLE cases have been reported, where HCC was in part supplied by the right IPA [4,7,8]. Shunting between the IPA and the pulmonary venous systems was not detected; thus, direct embolization is an unlikely source of brain emboli. Indirect evidence from extensive lipiodol embolization in both lungs indicates that simultaneous systemic and pulmonary shunts lead to non-target embolization. On color Doppler sonography, dilated vascular channels were seen inside the tumor. Although the initial angiogram could not demonstrate apparent AV shunting, a low volume shunt might have gone undetected pre-embolization. It is less likely but also possible that embolization altered the flow dynamics and opened up novel shunts inside the tumor.

Additional risk factors of CLE can be determined by comparing previously reported cases with the present one. Based on the literature the volume of lipiodol appears to correlate directly with the duration of post embolization recovery, and the complication rate increases when >20 mL is injected [10]. Due to the large size of the tumor, we used 50 mL lipiodol for the embolization, twice the standard dose. This could have significantly contributed to the extensive non-target embolization in the current case. In their review Wu *et al*. reported that nine out of 12 CLE cases occurred after repeated embolization sessions [7]. Prior embolizations of the feeding arteries could have resulted in extensive necrosis and collateralization of the vascular supply; thus, high flow AVS may have developed in the previously treated areas. No prior embolization was performed in our case. A patent foramen ovale (PFO) has a 10% to 20% prevalence in the adult population. This makes PFO a potential embolization pathway in CLE patients. Indeed, there are well documented CLE cases, when a PFO was verified with trans-esophageal echocardiography [8]. Still, in most of the CLE patients there is no evidence of intracardiac shunting [8]. The autopsy could not identify an ISD in the present case either.

## Conclusions

In summary CLE is a relatively rare, but well-documented complication of TAE in HCC patients. Cerebral embolization could be traced back to a pulmonary AV shunt in the current case, and it may have been a potential embolization source in other published cases, as well. Thus, in patients with advanced tumors and high risk of CLE a less aggressive embolization strategy or alternative treatment methods should be considered. If CLE still occurs, neurological symptoms may regress spontaneously, and most patients require symptomatic management only.

## Consent

Written informed consent was obtained from the patient.

## Competing interests

The authors declare that they have no competing interests.

## Authors’ contributions

Clinical studies and patient management discussed in the manuscript were performed by ZB, GL, GS, GV, PB, IK, and BF, the manuscript text was prepared by PNK, ZB, GL, and VB. Both VB and PNK were involved in the final text editing. VB approved the manuscript and supervised the whole study. All authors read and approved the final manuscript.
